# Multivariate Analyses of Amyloid-Beta Oligomer Populations Indicate a Connection between Pore Formation and Cytotoxicity

**DOI:** 10.1371/journal.pone.0047261

**Published:** 2012-10-15

**Authors:** Panchika Prangkio, Erik C. Yusko, David Sept, Jerry Yang, Michael Mayer

**Affiliations:** 1 Department of Biomedical Engineering, University of Michigan, Ann Arbor, Michigan, United States of America; 2 Center for Computational Medicine and Bioinformatics, University of Michigan, Ann Arbor, Michigan, United States of America; 3 Department of Chemistry and Biochemistry, University of California San Diego, La Jolla, California, United States of America; 4 Department of Chemical Engineering, University of Michigan, Ann Arbor, Michigan, United States of America; University of Akron, United States of America

## Abstract

Aggregates of amyloid-beta (Aβ) peptides are thought to be involved in the development of Alzheimer’s disease because they can change synaptic plasticity and induce neuronal cell death by inflammation, oxidative damage, and transmembrane pore formation. Exactly which oligomeric species underlie these cytotoxic effects remains unclear. The work presented here established well-controlled aggregation conditions of Aβ_ 1–40_ or Aβ_1–42_ peptides over a 20-day period and characterized these preparations with regard to their β-sheet content, degree of fibril formation, relative abundance of various oligomer sizes, and propensity to induce membrane pore formation and cytotoxicity. Using this multivariate data set, a systematic and inherently unbiased partial least squares (PLS) approach showed that for both peptides the abundance of oligomers in the tetramer to 13-mer range contributed positively to both pore formation and cytotoxicity, while monomers, dimers, trimers, and the largest oligomers (>210 kDa) were negatively correlated to both phenomena. Multivariate PLS analysis is ideally suited to handle complex data sets and interdependent variables such as relative oligomer concentrations, making it possible to elucidate structure function relationships in complex mixtures. This approach, therefore, introduces an enabling tool to the field of amyloid research, in which it is often difficult to interpret the activity of individual species within a complex mixture of bioactive species.

## Introduction

Alzheimer’s disease (AD) is characterized by the accumulation of amyloid-b (Aβ) peptide aggregates and the formation of insoluble plaques in the brain of affected patients [1]–[3]. The major components of these amyloid plaques are Aβ peptides with 40 (Aβ_1–40_) and 42 amino acids (Aβ_1–42_), both of which are thought to play an important role in AD pathogenesis [4]–[9]. The pathogenic mechanisms leading to AD are, however, not well understood and several hypotheses are being actively investigated [10]–[13]. These hypotheses are based on evidence that oligomeric Aβ peptides can change synaptic plasticity [9], [14]–[20] or cause neurotoxicity [21] by triggering inflammatory responses, oxidative damage [9], dysregulation of ion homeostasis (including Ca^2+^ ions) [2], [22], and altered kinase and phosphatase activities that can lead to neurofibrillary tangles [23]–[25].

With regard to the sizes of Aβ oligomers that might be the most important for the pathogenesis of AD, several in vitro neurotoxicity [26] and in vivo studies in mouse models of AD [27] implicated the following oligomers: dimers [28]–[34], trimers [35], tetramers to 9-mers (also called Aβ-derived diffusible ligands, ADDLs, with an estimated mass of 17–42 kDa) [16], [36]–[39], 12-mers [15] also called Aβ*56 [39], [40], and protofibrils containing aggregates larger than 100 kDa, which correspond to 22-mers and bigger aggregates [41]–[44]. In addition to these oligomeric species, several studies have revealed that insoluble Aβ fibrils induce neurotoxicity and impair synaptic transmission [21], [45]–[47].

Pore formation by Aβ is one plausible mechanism for toxicity and for antimicrobial peptide (AMP) activity [48], since oligomeric Aβ binds to lipid membranes [49], [50] and since Aβ-induced pores could result in aberrant flux of Ca^2+^ ions and cause cell death [6], [22], [51]–[55]. For instance, Soscia et al. showed recently that Aβ acts as an AMP against eight clinically relevant microorganisms and that brain homogenates of AD patients were significantly more antimicrobial than homogenates from age-matched control samples [48]. Various biochemical, biophysical, and computational techniques have indicated a range of aggregated Aβ species that could potentially induce pores or form ion channel-like structures in artificial lipid bilayers and in neuronal membranes [54], [56]–[59]. For instance, Jang et al. performed molecular dynamics (MD) simulations and proposed that 16- to 24-mers of Aβ arrange into pore-like structures [60], which are compatible with the dimensions and shape of putative Aβ pore structures obtained from atomic force microscopy (AFM) [61], [62]. In two separate MD studies, Strodel et al. identified tetramers or hexamers as the most stable structures that could form Aβ pores [63], while Shafrir et al. suggested that Aβ pores contain assemblies of six hexamers (i.e. a 36-mer) [64]. Based on mass spectrometry, Pan et al. recently revealed β-barrels of tetrameric Aβ_1–40_ peptides arranged in antiparallel β-turn-β motifs [65]. Demuro et al. demonstrated on Xenopus oocytes that Aβ_1–42_ oligomers in the range from 5- to 40-mers caused Ca^2+^ flux that was independent of endogenous ion channels [44]. Schauerte et al. combined single molecule fluorescence microscopy with ion current recordings and reported that hexamers were the smallest oligomeric Aβ structure that could permeabilize lipid membranes, while 12- to 14-mers resulted in pores with the largest conductivity for ions [66]. This work is particularly relevant since it was carried out with Aβ concentrations in the nanomolar range and, therefore, close to the concentrations of Aβ observed in human brains [67]. In addition, assays with liposomes showed dye leakage in the presence of Aβ oligomers and reduced leakage in the presence of fibrils [49]. Laganowsky et al. showed recently that a segment of the amyloid-forming protein αβ crystallin can form cylindrical barrels with an open central channel from six antiparallel proteins and that this so-called cylindrin structure is compatible with a sequence element from Aβ [68]β Lashuel et al. showed by electron microscopy that a mutant version of Aβ that is associated with early-onset Alzheimer’s disease, called Aβ_ARC_, formed pore-like assemblies from 40 to 60 monomers [69], while recent X-ray diffraction and electron microscopy studies showed that these pore-like assemblies may be formed by 20-mers of Aβ_1–42_ with cross-β architecture [70].

Here, we established a well-controlled and reproducible preparation method to form Aβ aggregates over 0, 1, 2, 3, 10 and 20 days in water. We examined the ability of these populations to form ion pores in planar lipid bilayers and separately quantified their cytotoxic effects on a human neuroblastoma cell line. For each time point during the aggregation process, we quantified the relative abundance of different Aβ oligomers and correlated individual oligomer levels with pore formation and toxicity data as well as with β-sheet content and degree of fibrillization. We carried out all assays with the same Aβ preparation at each time point in order to enable a systematic study of cross-correlation of abundance of oligomer species in two Aβ preparations (Aβ_1–40_ and Aβ_1–42_) with the propensity of the same preparations to form pores and cause cell death. To make this analysis quantitative, we adopted a partial least squares (PLS) approach and found that cytotoxicity and pore formation have identical dependencies on the oligomer distribution, supporting a causative relationship between these phenomena.

## Results and Discussion

### Probability of Pore Formation is Maximal after Incubating Aβ Samples for 2–3 Days

**Figure 1:**
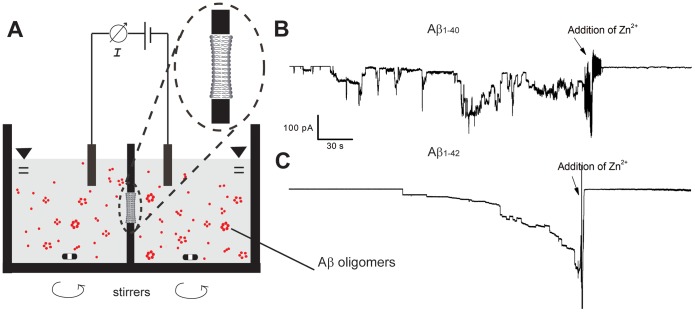
Pore formation by Aβ_1–40_ and Aβ_1–42_ in planar lipid bilayers. **A)** Cartoon of the experimental setup. **B)** Example of transmembrane ion flux induced by 15 µM Aβ_1–40_ prepared by one day incubation in water (method A, Table S1). **C)** Example of transmembrane current induced by 15 µM Aβ_1–42_ prepared by three day incubation (method A). Addition of 10 mM Zn^2+^ (arrows) inhibited Aβ-induced ion flux.

Figure 1 illustrates that both Aβ_1–40_ and Aβ_1–42_ caused channel-like ion flux across planar lipid bilayers that could be inhibited by Zn^2+^ ions, as reported previously [51], [53], [54], [61], [62], [71]–[78]. The conductance values of these recorded current modulations were not well defined and typically ranged from 80 pS to 0.8 nS, while their open pore lifetimes ranged from milliseconds to several minutes as reported previously [79]. These biophysical characteristics of Aβ-induced transmembrane current flux suggest that Aβ may either self-assemble to a transmembrane protein pore, reminiscent of ion channel proteins [71] or Aβ may interact with lipid membranes [49], [50], [80] and induce defects in the membrane, reminiscent of certain antimicrobial peptides [48], [54], [67]. It is also possible that both mechanisms act in parallel. In the context of this work, we chose the terminology “Aβ-induced ion flux” or “pore formation” in order to include both possibilities.

Since aggregation conditions affect the aggregation kinetics and potentially the morphology of Aβ aggregates, we compared five different aggregation methods of Aβ samples from various suppliers for their ability to form pores in planar lipid bilayers (Supporting Information, Table S1, Methods A–E and Figure S1, S2, and S3). Among these methods, we selected incubation of Aβ samples in water for 0 to 20 days (Method A) because it made it possible to study the effects of Aβ aggregation on pore formation over time while being compatible with assays for cytotoxicity, CD spectroscopy, and ThT fluorescence assays (see Supporting Information Section S2.2 for a comparison and discussion of these five methods for Aβ aggregation).

**Figure 2:**
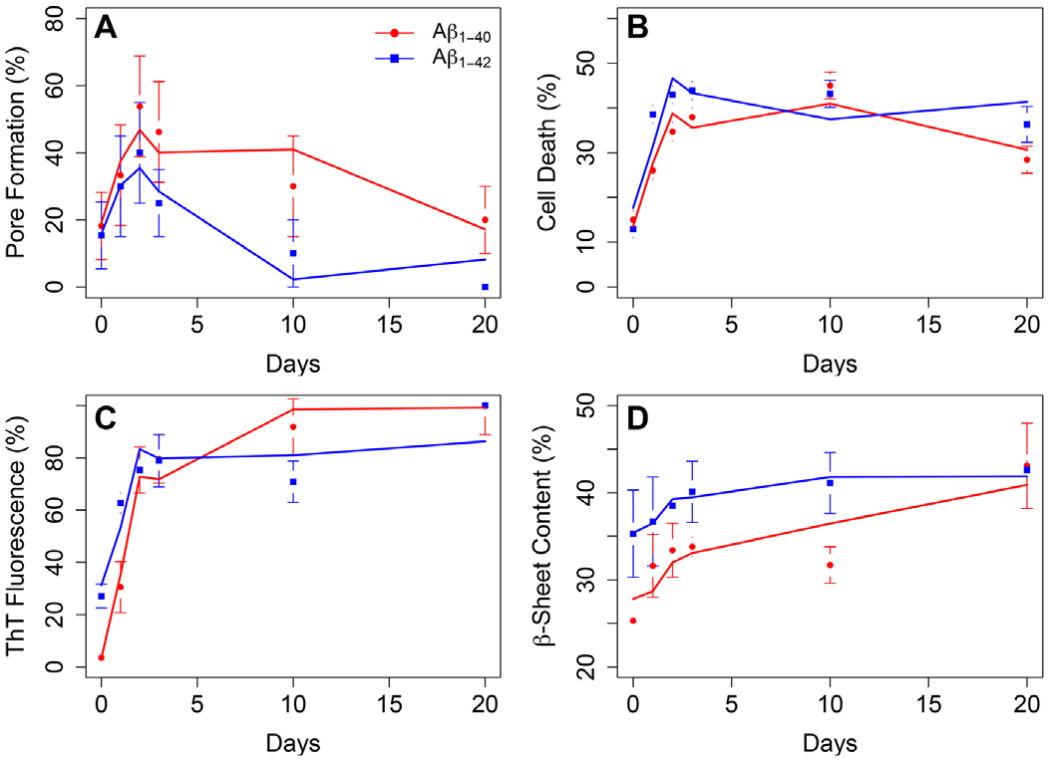
Pore formation, cytotoxicity, ThT fluorescence and β-sheet content as a function of aggregation time of Aβ samples in water (method A). Red and blue curves in each panel are the two component predictions from PLS regression for Aβ_1–40_ or Aβ_1–42_, respectively. **A)** Percentage of experiments that showed pore formation in planar lipid bilayers. Each point represents 10–15 experiments in the presence of 15–25 µM Aβ_1–40_ or Aβ_1–42_; error bars represent the error of proportion. **B)** Cell death of human neuroblastoma SH-SY5Y cells 24 h after exposure to serum-free media containing 20 µM Aβ prepared by method A. Each point represents 5–15 independent experiments. **C)** Degree of fibril formation as determined by ThT fluorescence. Each point represents an average from 5–20 experiments, error bars represent standard errors of the mean. **D)** Beta-sheet formation as determined by CD spectroscopy. Each point represents an average from 3–5 experiments, error bars represent standard errors of the mean.

Fig. 2A shows that the probability of pore formation was highest when Aβ samples were pre-incubated in water for 2–3 d in the case of Aβ_1–40_ or for 2 d in the case of Aβ_1–42_. In contrast, pore formation was least likely when Aβ samples were pre-incubated for 0 d or 20 d. With regard to the biophysical characteristics of Aβ-induced ion flux, we did not observe consistent differences in the conductance or lifetime of ion flux events among different aggregation methods (A–E, Table S1). In fact, the conductance and lifetime of Aβ-induced pores varied significantly between experiments with the same preparation method or within the 30 min of one experiment. This variability suggests that Aβ-induced pores are dynamic structures with a range of conformations and sizes [79]. Control experiments without Aβ revealed ion flux in one of eleven experiments; this activity was likely due to a sporadic instability of bilayers within the 30 min recording period; these instabilities can occur during bilayer recordings [81].

### Cytotoxicity is Maximal after Aggregating Aβ_1–40_ for Three to Ten Days and Aβ_1–42_ for Two to Ten Days

Samples of Aβ resulted in maximal cytotoxicity in a human neuroblastoma cell line after incubating Aβ_1–40_ for three to ten days, while the toxicity of Aβ_1–42_ was highest between two and ten days (Fig. 2B). Wogulis et al. found a similar time-dependent trend of toxicity albeit under different conditions and therefore different kinetics [82]. For information on the toxicity of all Aβ preparation methods explored here, see Table S1.

### Oligomer Levels during Aggregation

**Figure 3:**
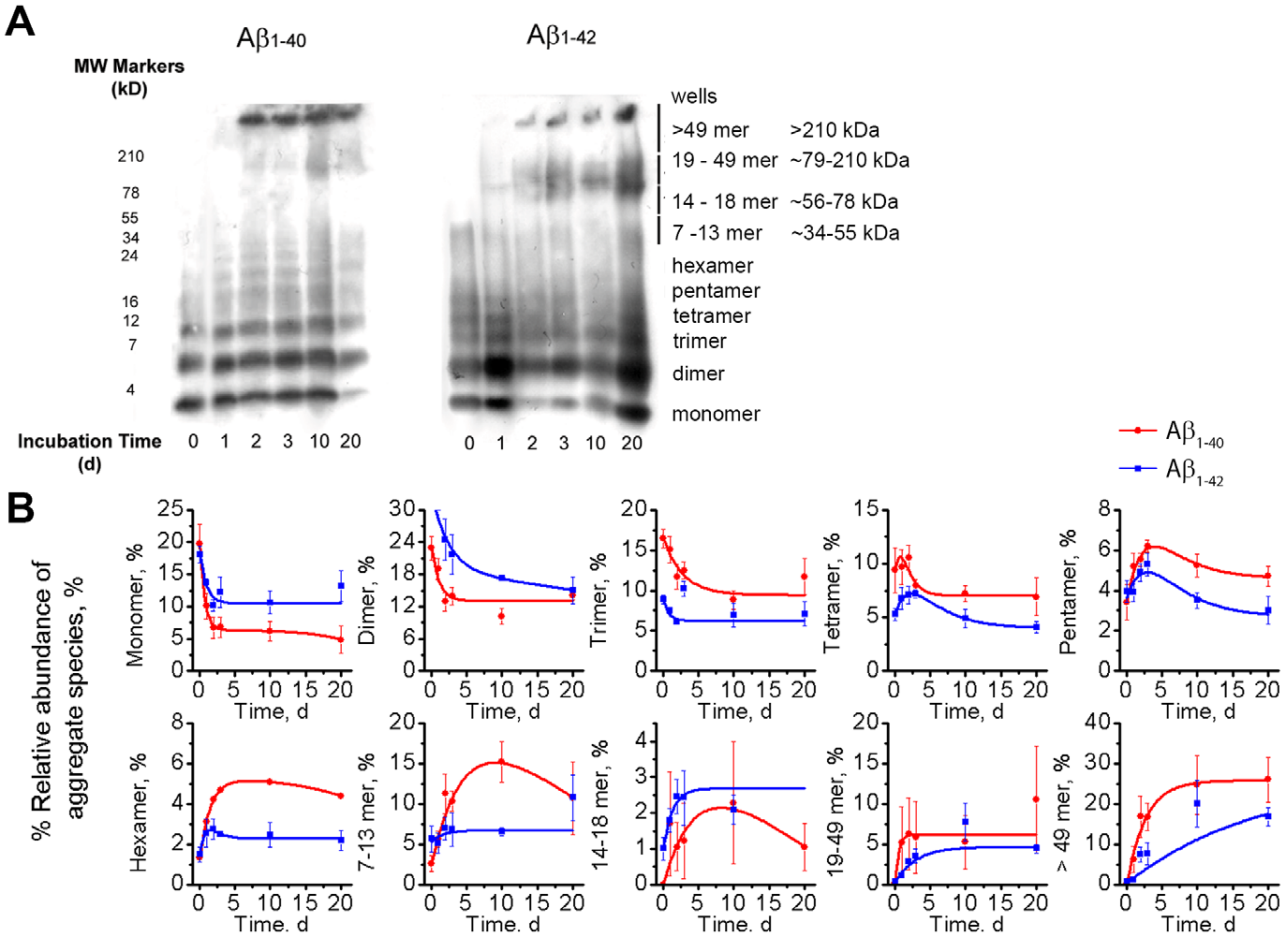
Separation of Aβ samples by SDS-PAGE followed by Western blot and densitometry analysis. **A)** Example of a Western blot of after SDS-PAGE of Aβ_1–40_ (left) and Aβ_1–42_ (right) preparations that had been incubated in water for 0, 1, 2, 3, 10, and 20 days (method A). All samples were cross-linked with 12 mM glutaraldehyde before electrophoresis through a 16.5% Tris-Tricine gel. Grouping of oligomers is indicated on the right. **B)** Average relative abundance of aggregated Aβ_1–40_ (red) and Aβ_1–42_ (blue) species of different size as a function of aggregation time. Each point represents the mean value of the relative abundance of each species from 4 to 6 gels; error bars represent the standard error of the mean. Red and blue curves are best curve fits of equation S1 to the data (see Supporting Information).

In order to correlate both pore formation and cytotoxicity with the aggregation state of Aβ samples, we determined the relative abundance of monomeric and oligomeric species of Aβ as a function of incubation time in water. Choosing among the available analytical methods, which all have their limitations [67], we selected SDS-PAGE separation of cross-linked Aβ samples combined with Western blotting (Fig. 3) and densitometry for this quantification (i.e., analysis of grey levels from scanned images of Western blotted gels). This approach yielded the resolution of individual oligomer species or groups of oligomers as indicated in Fig. 3A (see Supporting Information, Section S2.4 and S2.5 for a discussion on using Western blot for this analysis).

Figure 3B shows that the relative abundance of low molecular weight aggregates (i.e., monomers to trimers) decreased as a function of aggregation time, whereas the relative abundance of intermediate-sized oligomers (tetramers to 13-mers in the case of Aβ_1–40_ and tetramers to hexamers in the case of Aβ_1–42_) went through a maximum after approximately 2 to 3 days. We observed, as expected, that the relative abundance of high molecular weight oligomers (>18-mers) gradually increased over time.

Since the binding of anti-Aβ antibodies to oligomers could be conformation dependent, we compared the results from densitometry analysis after Western blot with densitometry analysis after silver staining [83]. Results with Aβ_1–40_ showed that both detection techniques revealed similar trends for the change of relative abundance of small oligomer sizes but quantification of abundance for aggregates larger than hexamers was difficult with silver staining, as reported previously (see *Supporting Information*, Fig. S4, and Fig. S5) [83], [84]. Silver staining results suggested very low relative amounts of these larger aggregates despite results from TEM analysis, which showed the presence of a significant fraction of Aβ protofibrils with lengths above 20 nm in three day samples and in samples with longer aggregation time (see Supporting Information, Figure S6 and Section S2.5). Therefore, we performed the quantitative analysis of oligomer abundance in this work based on Western blots. The time-dependent variations of the relative amounts of individual Aβ species as determined by SDS-PAGE (Fig. 3) indicate that this Western-blot-based comparative analysis among differently-aged Aβ preparations did indeed reveal information about the kinetics of aggregation. Possible artifacts by SDS-induced aggregation [67] or excess aggregation induced by chemical cross-linking [85], if present, were not sufficiently large to mask this time dependence. In fact, Ono et al. showed that purified samples of cross-linked Aβ oligomers changed their composition by less than 15% during analysis by SDS-PAGE [23].

### Characterization of Aβ Aggregation by Thioflavin T Fluorescence and Circular Dichroism

We used thioflavin T (ThT) fluorescence as a surrogate to monitor fibrillization [23] and measured circular dichroism (CD) spectra to examine the extent of secondary structure in the samples of Aβ_1–40_ and Aβ_1–42_ in water after pre-incubation times of 0, 1, 2, 3, 10, and 20 d. The ThT signal from Aβ_1–40_ samples reached its half-maximal signal between one and two days (τ_½max_ = 1.6 d) and increased only slowly after the third day (Fig. 2C). In case of Aβ_1–42_, the half-maximal ThT signal was reached in less than one day (τ_½max_ = 0.9 d). Data from CD spectroscopy revealed that the relative amount of β-sheet followed a similar trend as the ThT signal, i.e., a rapid increase during the first 1–2 days, followed by a slower increase as aggregation continued (Fig. 2D and Supporting Information, Fig. S7 and Table S2). Together, these ensemble measurements suggested a good correlation between toxicity, ThT fluorescence, and β-sheet content, as reported previously by Simmons et al [86], while pore formation correlated only during the first three days with ThT fluorescence and β-sheet content. The most pore-forming and most toxic Aβ preparations were those whose ThT and CD signal transitioned from the initial fast increase to the phase of slow increase.

### Dependence of Pore Formation and Cytotoxicity on the Oligomer Populations

In order to develop a model that describes the effect of oligomer populations on pore formation and cytotoxicity, we adopted a multivariate regression approach. This approach is particularly well suited here since we had observations at six time points over twenty days that included the measured levels of pore formation, cytotoxicity, β-sheet content, and ThT fluorescence, as well as the relative abundance of nine different oligomer species for two Aβ peptides. A multiple linear regression approach might seem logical in this case, but was not appropriate given the relative uncertainties in the explanatory variables (oligomer levels) and response variables (pore formation, cytotoxicity, ThT fluorescence, and β-sheet content) as well as the significant correlations in the oligomer population. Instead, we used a partial least squares (PLS) formalism since it is suited for limited data sets and correlated predictor variables, and has been shown to work well for similar problems ranging from pharmaceutics to neuroimaging [87]–[89]. Further, PLS regression does not require any assumptions about the relationship between the observations and the predictor variables, and is ideal for mixtures of species whose predictor variables (i.e., the relative abundance of each oligomer class) contain uncertainty and are interdependent (i.e., tetramer levels depend on trimer and pentamer levels and on the levels of all other oligomer classes in the preparation).

We performed PLS regression using the oligomer abundance as predictor variables and simultaneously carried out regression for all four observables: pore formation, cytotoxicity, β-sheet content, and ThT fluorescence. We carried out this analysis twice by treating data from Aβ_1–40_ and Aβ_1–42_ independently and found for each case that a model with two components or latent variables [87] was sufficient to explain 92–95% of the variance of the four observables (see Fig. S8). Interestingly, the component vectors that were obtained for Aβ_1–40_ and Aβ_1–42_ are nearly identical, suggesting that the dependence of pore formation, cytotoxicity, β-sheet content, and ThT signal on the underlying oligomer distributions must be similar for both peptides.

**Figure 4:**
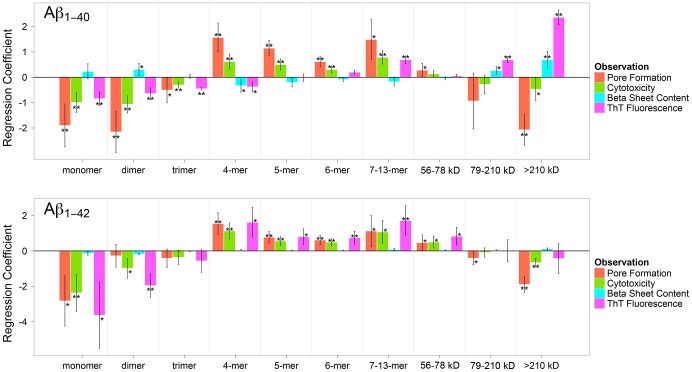
Dependence of pore formation, cytotoxicity, β-sheet content and ThT fluorescence on the oligomer levels of Aβ_1–40_ (top) and Aβ_1–42_ (bottom). The sign of the regression coefficients indicates whether a particular species contributes positively or negatively to a particular observation. The error bars are the standard deviation of the coefficient estimated through a jackknife procedure [90]. Coefficients less than 0.25 were considered insignificant and values that were at least one or two standard deviations from zero are marked as significant (*) or highly significant (**) respectively.

Figure 4 shows the PLS regression coefficients for all four observables for Aβ_1–40_ and Aβ_1–42_ peptides. In this plot, positive or negative coefficients indicate a positive or negative effect on the given observation, and the asterisks indicate the statistical significance of a given coefficient based on jackknife test [90]. Again we found that both peptides had a nearly identical dependence on the distribution of oligomeric species. In terms of specific observations, cytotoxicity and pore formation had highly correlated dependencies: both had a significant negative contribution from small oligomers (monomers to trimers), significant positive contributions from tetramers to hexamers as well as the 7- to 13-mer group, and then again negative contributions from large oligomers, particularly the >210 kDa class of protofibrils.

These results agree well with previous observations, in particular that the smallest oligomers may in fact be protective rather than pathologic [24], [55], [91], [92], and that oligomers in the tetramer to 13-mer range exert the most significant effect on Aβ-induced pore formation and toxicity [26], [39], [44], [49], [58], [61]–[66]. For instance, Lin et al showed that at least three monomers are required to form Aβ-induced pores [93], while Pan et al. specifically identified a porin-like, tetrameric β-barrel oligomer in solution and proposed that it may form ion pores in membranes [65]. Moreover, a recent single molecule microscopy study showed that the dimer to tetramer range of Aβ_1–40_ constitutes the majority of species binding to neuronal cells at physiological Aβconcentration and that these binding events were associated with sporadic Ca^2+^ leakage [94]. Bernstein et al. found by ion mobility coupled with mass spectrometry that Aβ_1–40_ forms stable annular tetramers, while Aβ_1–42_ forms quasiplanar, annular hexamers as well as dodecamers (from stacking two such hexamers) [39]. Annular oligomers were proposed before as possible pore-forming Aβ structures [61], [68], [69], [95]. Schauerte et al. combined bilayer experiments with single molecule fluorescence studies and reported that hexamers to 14-mers of Aβ formed pores [66], while Demuro et al. showed by Ca^2+^-imaging that 5- to 40-mers of Aβ induced pores for Ca^2+^ in plasma membranes [44]. It is surprising and revealing that PLS analysis on the results from planar lipid bilayer experiments carried out here under significantly different experimental conditions identified the same range of oligomer sizes as pore-forming as those identified by single molecule studies on live cells [44], [94]. This agreement between different techniques suggests that similar Aβ pore structures can form in artificial lipid bilayers and in cellular membranes. Evidence from electrophysiological [44], [51], [54], [96], electron microscopic [69], [70], X-ray diffraction [70], NMR [97], [98], AFM [56], [61], mass spectrometry [39], [65], and molecular dynamics experiments [97], [99], [100] suggests that these Aβ pore structures are formed by an annular arrangement of several monomers.

In order to further examine the plausibility for the formation of these proposed Aβ pore structures, we applied a simple barrel-stave model of such an Aβ pore to estimate the numbers of monomers per pore from single channel recordings of Aβ-induced ion flux (Fig. 1) and compared these numbers with the results from PLS analysis. This analysis revealed that the theoretically expected single channel conductance values through the lumen of Aβ pores that form by assembly of four to thirteen Aβ monomers to a regular polygon of transmembrane cylinders range from 47 pS to 1.2 nS (Fig. S9, Supporting Information). This range is in excellent agreement with the experimentally measured range of 80 pS to 0.8 nS and illustrates that annular arrangements of four to thirteen Aβ monomers could indeed form ion pores in a size range that is consistent with experimentally measured values of single channel conductance (Fig. S9). In contrast, trimeric assemblies as well as assemblies of more than thirteen Aβ monomers would have predicted conductance values that are outside of the majority of experimentally measured values according to this simple model (Fig. S9).

The size range from dimers to 8-mers constitutes the predominant Aβ oligomer fraction in the brain [15] and includes the range of oligomer sizes suspected to be the most clinically relevant in the context of Alzheimer’s disease [23]. Injection of purified 9- and 12-mers into the ventricle of pre-trained rats, for instance, had acute effects on special memory performance [15]. Finally, Ono et al. reported toxicity studies with cross-linked and purified Aβ oligomer species [23]. These studies showed the following rank order of increasing and additive toxicity: Aβ monomers < dimers < trimers < tetramers (larger aggregates were not examined).

Unlike the tetramer to 13-mer range of oligomers, the monomer, dimer, and trimer species were anticorrelated with both pore formation and cytotoxicity (Fig. 4). These findings agree with observations by Schauerte et al. that dimers do not permeabilize membranes and with previous observations that at least three peptides are required to form a pore [66], [79], [93]. These results deviate, however, from reports that Aβ dimers are the most toxic species [28]–[34]. Belinova et al. recently reviewed these findings and concluded that the identified dimers may aggregate to larger oligomers during toxicity assays and that the observed toxicity may have originated from these larger species [67]. In fact, O’Nuallain confirmed rapid aggregation of these dimers to metastable protofibrils [31].

The observation that large oligomers (>210 kDa) are anticorrelated with pore formation and toxicity, rather than making no contribution, may be explained by recent findings that monomers bind to and dissociate from Aβ oligomers and fibrils in a dynamic process [23], [101], [102]. Therefore, fibrils (and, presumably, large oligomers) can act as non-toxic scavengers of pore-forming and toxic Aβ species [67] that may otherwise be free to form small toxic oligomers [27], [103]. Several recent reports showed that Aβ fibrils are not toxic [13], [23], [58], [103]–[105]. This close correlation – both, negative and positive – between pore formation and cytotoxicity over the entire size range of both Aβ peptides implies a close mechanistic connection and is consistent with recent findings by Diaz et al. that blockers of Aβ “ion channels” protected cells from Aβ-induced toxicity [106].

Both ThT fluorescence and β-sheet content have little contribution from small and intermediate oligomers in these ensemble measurements – all the coefficients are relatively small and only a few are significant, however, in the case of Aβ_1–40_, both of these observables have strong, positive contributions from the 78–210 kDa and >210 kDa oligomer classes. As one might expect, the β-sheet and ThT signals from these large oligomers are largely correlated, however both observables are anticorrelated with pore formation and cytotoxicity. This result is important since it demonstrates that fibrillization, β-sheet content, pore formation, and toxicity can depend differently on various oligomer species (Fig. 4), although ensemble measurements on mixtures of Aβ species suggest the misleading result that all four observables are correlated with each other (Fig. 2).

### Conclusions

One of the proposed mechanisms of Aβ-induced neurotoxicity is pore formation [69], [71], [107]. Studies from different research groups using various techniques [51]–[54], [56], [58]–[64], [66], [108] have indicated that aggregated forms of Aβ with a broad range of sizes can induce ion flux through artificial and cellular lipid membranes. One of the big challenges for revealing the pathogenic mechanisms of Aβ is that measurements are performed on a dynamic and distributed ensemble of oligomers [12], [109], [110]. In fact, it is intrinsically difficult or impossible to perform assays on individual Aβ species because even cross-linked and purified Aβ dimers were reported to form a small fraction of tetramers [23] or larger oligomers during the course of experiments [67], [109], [110]. Moreover, assays performed on these complex mixtures of Aβ oligomers are usually limited to bulk measurements whose read-outs such as cytotoxicity, extent of fibrillization, or β-sheet content may appear to be correlated (Fig. 2), while the biophysical basis for these phenomena may depend on different oligomer species in the mixture (Fig. 4) [67], [80]. In order to tackle these complications, we used a PLS formalism that reduced the dimensionality from ten oligomer classes to two component vectors, and made it possible to analyze four observables at six time points simultaneously over a 20 day period. This analysis showed that measures of structure such as β-sheet content or degree of fibrillization depended on different Aβ oligomer species than pore formation and toxicity. Interestingly, pore formation and toxicity showed nearly identical dependence on different species in the oligomer distribution. Moreover, the close connection between pore formation and toxicity was observed independently for Aβ_1–40_ and Aβ_1–42_ despite differences in aggregation kinetics (Fig. 2) and relative amounts of aggregated Aβ species of different size at any given time point (Fig. 3) in these two amyloid preparations [39].

Based on this analysis, we found tetramers to 13-mers contributed positively to pore formation and toxicity and within this range, the tetramer to hexamer populations showed the highest statistical significance for both peptides. This size range is in excellent agreement with previously identified size ranges of Aβ oligomers as determined by biophysical, computational, and structural studies on pore formation as well as with in-vitro and in-vivo studies of neurotoxicity.

Multivariate PLS analysis also revealed that the smallest (monomer to trimer) and largest Aβ species (>210 kDa) contributed negatively to pore formation and cytotoxicity and may therefore act as inert sinks that scavenge toxic oligomers [67]. Taken together, these results provide evidence that – under the experimental conditions explored in this work – pore formation was a significant component of cytotoxicity. The tetrameric to hexameric oligomers that we identified as the most pore-forming and toxic oligomers, have been found in the brain of AD patients and it is plausible that they could cause sporadic leaks of calcium ions into neurons [44], [94]. These insults, over years of exposure, may contribute to cumulative neuronal damage in Alzheimer’s disease.

This study introduces an unbiased, systematic approach to deduce statistical correlations between the size distribution of Aβ oligomers and pore formation, toxicity, β-sheet content and extent of fibrillization. This multivariate approach is different from previous studies on Aβ-induced toxicity and pore formation, nonetheless its results agree with many previous findings on Aβ-induced pore formation or cytotoxicity. This approach therefore supports the idea that Aβ-induced pore formation may be a significant mechanism of its toxicity. Similar multivariate PLS analyses might be valuable for other amyloidogenic diseases in which standard ensemble measurements often mask the cytotoxic contributions of individual aggregate species in complex and dynamic mixtures of amyloids.

## Materials and Methods

Please refer to the Supporting Information for details on chemicals, materials and methods such as formation of lipid bilayers, current recordings, preparation of AAβ samples, toxicity assays, Western blotting, silver staining, densitometry analysis, ThT fluorescence assays, CD spectrometry, transmission electron microscopy, statistical analysis, partial least squares analysis as well as for Supplementary Results, Tables, and Figures.

## Supporting Information

Figure S1 ***SDS-PAGE/Western blot of Aβ samples from different suppliers with or without treatment with HFIP followed by lyophilization.*** All Aβ samples were prepared freshly in deionized H_2_O at a conc. of 1 mg mL^−1^. Each well in the 18%Tris-HCl gel (Bio-rad) was loaded with 0.2 µg of Aβ. Lane 1 = Bachem (non-lyophilized); 2 = GL Biochem, Ltd (Shanghai) (non-lyophilized); 3 = Biopeptide Inc. (non- lyophilized); 4 = GL Biochem, Ltd (Shanghai) (lyophilized); and 5 = Biopeptide Inc. (lyophilized). Aggregation of Aβ varies in commercial sources. HFIP treatment followed by lyophilization for 2 d removed all aggregates of Aβ larger than ∼ 12 kDa in the case of Aβ_1–40_ and removed large Aβ aggregates (>225 kDa) in the case of Aβ_1–42_.
(TIF)

Figure S2 ***19F-NMR spectroscopy of HFIP in CD3OD (left) and Aβ1–40 sample that was incubated with HFIP and then lyophilized for two days*** **as described in the Materials and Methods section (right).** ^19^F resonance of HFIP gave a doublet at −77.8 ppm, while the peak was absent after Aβ was lyophilized in HFIP for 48 h.
(TIF)

Figure S3 ***SDS-PAGE/Western blotting of cross-linked Aβ1–40 and Aβ1–42 samples from various preparation methods.*** **1)** Method B: non-HFIP treated Aβ (GL Biochem, Ltd) in di-H_2_O with 0 d incubation; **2)** Method C: Modified Kayed preparation; **3)** Method D: Aβ proteoliposomes containing DOPS; and **4)** Method E: Aβ proteoliposomes containing 30% of positively charged DOTAP lipids. The presence of high molecular weight oligomers from these four samples indicates that these preparations accelerated the aggregation of Aβcompared to preparations in diH_2_O (Method A) (e.g, lanes 4 and 5 in Fig. S1) [104], [111].
(TIF)

Figure S4 ***Silver staining after SDS-PAGE of cross-linked Aβ1–40 samples prepared by method A for 0 to 20 d.*** Two micrograms of sample were loaded into each well. The relative amount of intermediate aggregates (dimers to hexamers) or large aggregates of Aβ (the species in lane 10 and 20 in the stacking gel) increased with incubation time. The stacking portion of the silver stained gel appeared with a dark background even in the absence of protein (blank) as shown in the first lane on the left of the gel, making quantitative analysis of large aggregates by silver staining difficult.
(TIF)

Figure S5 ***Relative abundance of various cross-linked Aβ1–40 aggregates of different size obtained after SDS-PAGE and silver staining.*** The intensity on the gel was corrected by subtraction of the blank (in the absence of protein). Each point represents the average of relative abundance of aggregates from six independent gels and samples; error bars represent the standard error of the mean. Red curves are best fits of equation (S1) to the data.
(TIF)

Figure S6 ***Transmission electron micrographs of Aβ1–40 aggregates.*** Micrographs were taken of Aβ_1–40_ aggregates after the aggregates had been incubated for zero, one, two, or three days according to Method A. Two micrographs are shown for each day, each taken at different locations on the TEM grid.
(TIF)

Figure S7 ***Circular dichroism spectra of Aβ1–40 (left panel) and Aβ1–42 (right panel) samples prepared by method A as a function of incubation time.*** With increasing incubation time, the Aβ-sheet content in Aβ samples increased, while the random coil content decreased.
(TIF)

Figure S8 ***Loadings of the first and second components (latent variables) for both Aβ peptides as determined by PLS regression.*** The first components for Aβ_1–40_ and Aβ_1–42_ (solid lines) and the second components for Aβ_1–40_ and Aβ_1–42_ (dotted lines) are nearly identical and have inner products of 0.91 and 0.87, respectively. The percentages listed indicate the percent of the variance explained by each component. These results indicate that the relationship between the four observables and the oligomer ensemble must be similar for the two peptides.
(TIF)

Figure S9 ***Comparison of experimentally measured single channel conductance values of Aβ1–40 pores with theoretically predicted values.*** These box plots were constructed from the amplitude of single-step current jumps in current *versus* time traces such as those shown in Fig. 1 of the main text. The first six box plots (Aβ 40-0d to Aβ 40-20d) represent the distribution of single channel conductance values from Aβ preparations that had been pre-incubated for 0 to 20 days prior to planar lipid bilayer recordings under the same conditions as in Fig 1 of the main text. The last box plot (dark yellow) represents the theoretical estimate of single channel conductance values of a model of Aβ pores that assumes an annular assembly of Aβ monomers to a regular polygon with an internal pore lumen. In this model, each Aβ monomer is represented by a transmembrane cylinder with a diameter, *d*, of 1 nm (approximate diameter of a transmembrane peptide) [112] and a length, *l*, of 3.5 nm (approximate thickness of a lipid bilayer). The centers of the cross-section of each cylinder are located at the corners of the regular polygon and the lengths of the polygon sides are equal to *d*. The theoretically predicted box plot was constructed from estimated single channel conductance values of eleven possible regular polygons ranging from the smallest one (assembly of three monomers to a triangle) to a regular polygon of 13 monomers. We estimated the single channel conductance as a function of the number of Aβ monomers, *N*, in these assemblies by calculating the area of the lumen *A_L_*(*N*) in such a pore. We obtained this area by calculating the area of a regular polygon with *N* sides, *A_RP_*(*N*) and subtracting the area of circle sectors, *A_CS_*(*N*), that overlap with the polygon area. The area of a regular polygon is given by: *A_RP_*(*N*) = *d* ^2^ × *N*/[4 × tan(180°/*N*)] and the area of circle sectors depends on the angle, α, between the sides of a regular polygon – which is given by α (*N*) = 180° (*N* –2)/*N* – in the following way: *A_CS_*(*N*) = α (*N*) × π × *d* ^2^/(4 × 360°). Therefore, the area of the lumen is given by: *A_L_*(*N*) = *A_RP_*(*N*) – *N* × *A_CS_*(*N*) = (*d* ^2^/4) × [*N*/tan(180°/*N*) – (π/2) × (*N* –2)]. With *A_L_*(*N*), we calculated the area equivalent radius of a circle as a function of the number of monomers, *r*(*N*), i.e. *r*(*N*) = (*A_L_*(*N*)/π)^0^.^5^, which we then used to calculate the single channel conductance,γ(*N*), through a pore with radius *r*(*N*) and length *l* in an electrolyte with resistivity ρ using the relationship γ(*N*) = [(*l* + π × *r*(*N*)/2) ×ρ/[π × *r* ^2^(*N*)]]^−1^ [113]. Since we used a lipid bilayer with 50 mol% negatively-charged lipid head groups, we estimated the local resistivity near the membrane from work by Apell *et al* [114] and found ρ = 0.313 Ω m for the experimental conditions used in Fig. 1 of the main text. The predicted box plot (dark yellow) was then obtained from the γ(*N*) values of oligomers with 3 to 13 monomers, weighed by the relative abundance of each of these oligomers as shown in Fig. 3B of the main text. In these plots, the box encloses the 25^th^ to 75^th^ percentile, the whiskers enclose the 5^th^ to 95^th^ percentile, the crosses enclose the 1^st^ to 99^th^ percentile, the hyphens indicate the minimum and maximum single channel conductance value, while the horizontal line in the box indicates the median and the open square symbol indicates the mean single channel conductance value.
(TIF)

Table S1 **Comparison of different Aβ aggregation procedures with regard to the propensity of the resulting Aβ preparation to form pores in planar lipid bilayers and to kill cells.**
(DOCX)

Table S2 **Deconvolution of CD spectra of Aβin different preparation methods.**
(DOCX)
