# Supplementary figures and images for: Multivariate Analyses of Amyloid-Beta Oligomer Populations Indicate a Connection between Pore Formation and Cytotoxicity
Source: PLoS One. 2012 Oct 15;7(10):e47261. doi: 10.1371/journal.pone.0047261 (PMC3471831; doi:10.1371/journal.pone.0047261)

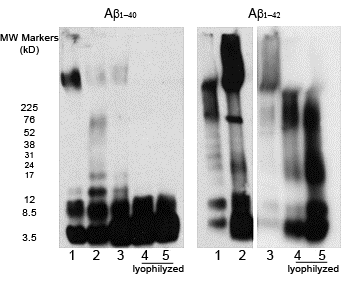

Supplement: Figure S1 — SDS-PAGE/Western blot of Aβ samples from different suppliers with or without treatment with HFIP followed by lyophilization. All Aβ samples were prepared freshly in deionized H2O at a conc. of 1 mg mL−1. Each well in the 18%Tris-HCl gel (Bio-rad) was loaded with 0.2 µg of Aβ. Lane 1 = Bachem (non-lyophilized); 2 = GL Biochem, Ltd (Shanghai) (non-lyophilized); 3 = Biopeptide Inc. (non- lyophilized); 4 = GL Biochem, Ltd (Shanghai) (lyophilized); and 5 = Biopeptide Inc. (lyophilized). Aggregation of Aβ varies in commercial sources. HFIP treatment followed by lyophilization for 2 d removed all aggregates of Aβ larger than ∼ 12 kDa in the case of Aβ1–40 and removed large Aβ aggregates (>225 kDa) in the case of Aβ1–42. (TIF) [file pone.0047261.s001.tif]

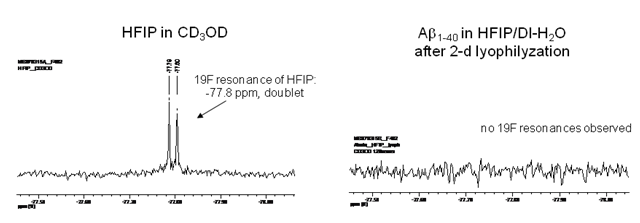

Supplement: Figure S2 — 19F-NMR spectroscopy of HFIP in CD3OD (left) and Aβ1–40 sample that was incubated with HFIP and then lyophilized for two days as described in the Materials and Methods section (right). 19F resonance of HFIP gave a doublet at −77.8 ppm, while the peak was absent after Aβ was lyophilized in HFIP for 48 h. (TIF) [file pone.0047261.s002.tif]

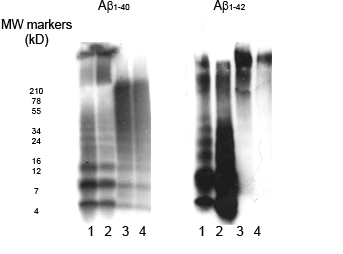

Supplement: Figure S3 — SDS-PAGE/Western blotting of cross-linked Aβ1–40 and Aβ1–42 samples from various preparation methods. 1) Method B: non-HFIP treated Aβ (GL Biochem, Ltd) in di-H2O with 0 d incubation; 2) Method C: Modified Kayed preparation; 3) Method D: Aβ proteoliposomes containing DOPS; and 4) Method E: Aβ proteoliposomes containing 30% of positively charged DOTAP lipids. The presence of high molecular weight oligomers from these four samples indicates that these preparations accelerated the aggregation of Aβcompared to preparations in diH2O (Method A) (e.g, lanes 4 and 5 in Fig. S1) [104], [111]. (TIF) [file pone.0047261.s003.tif]

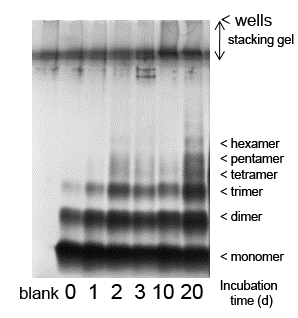

Supplement: Figure S4 — Silver staining after SDS-PAGE of cross-linked Aβ1–40 samples prepared by method A for 0 to 20 d. Two micrograms of sample were loaded into each well. The relative amount of intermediate aggregates (dimers to hexamers) or large aggregates of Aβ (the species in lane 10 and 20 in the stacking gel) increased with incubation time. The stacking portion of the silver stained gel appeared with a dark background even in the absence of protein (blank) as shown in the first lane on the left of the gel, making quantitative analysis of large aggregates by silver staining difficult. (TIF) [file pone.0047261.s004.tif]

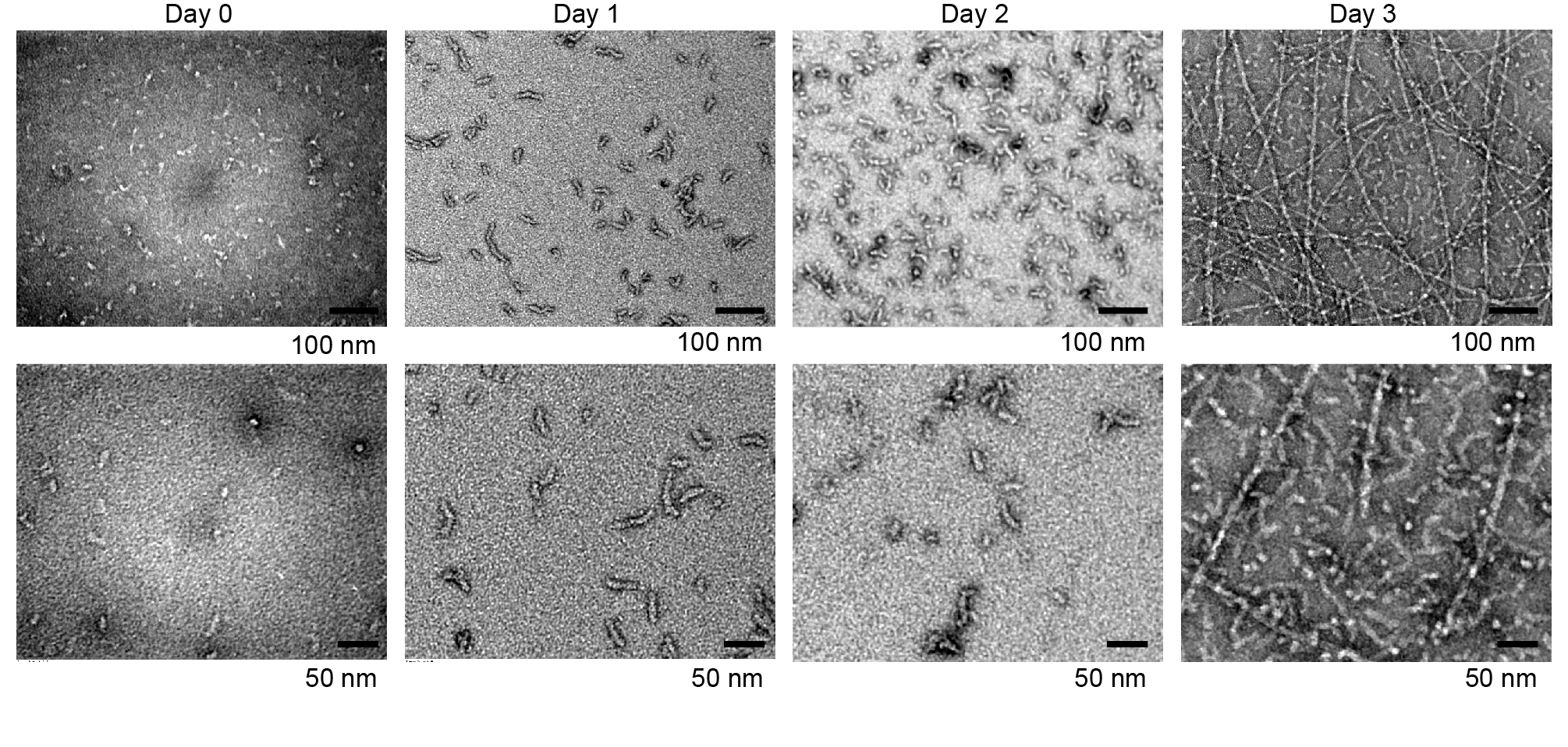

Supplement: Figure S6 — Transmission electron micrographs of Aβ1–40 aggregates. Micrographs were taken of Aβ1–40 aggregates after the aggregates had been incubated for zero, one, two, or three days according to Method A. Two micrographs are shown for each day, each taken at different locations on the TEM grid. (TIF) [file pone.0047261.s006.tif]

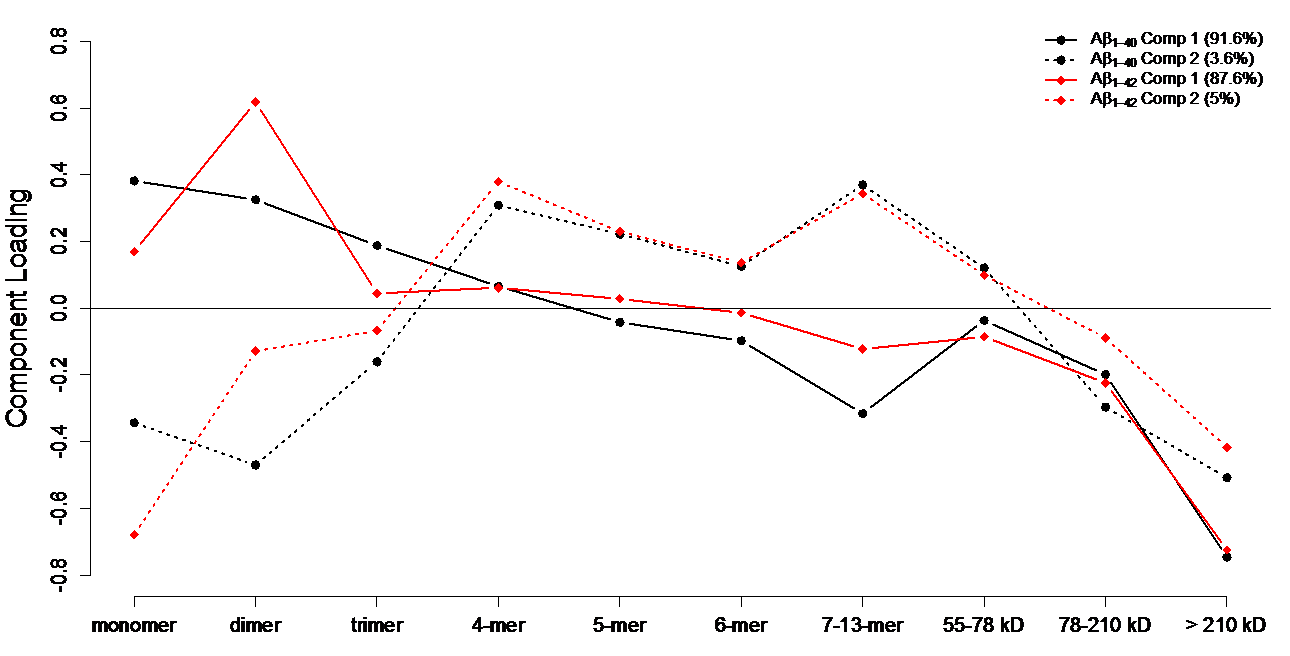

Supplement: Figure S8 — Loadings of the first and second components (latent variables) for both Aβ peptides as determined by PLS regression. The first components for Aβ1–40 and Aβ1–42 (solid lines) and the second components for Aβ1–40 and Aβ1–42 (dotted lines) are nearly identical and have inner products of 0.91 and 0.87, respectively. The percentages listed indicate the percent of the variance explained by each component. These results indicate that the relationship between the four observables and the oligomer ensemble must be similar for the two peptides. (TIF) [file pone.0047261.s008.tif]
