# Supplementary material for: Multivariate Analyses of Amyloid-Beta Oligomer Populations Indicate a Connection between Pore Formation and Cytotoxicity
Source: PLoS One. 2012 Oct 15;7(10):e47261. doi: 10.1371/journal.pone.0047261 (PMC3471831; doi:10.1371/journal.pone.0047261)
Supplement: Table S1 — Comparison of different Aβ aggregation procedures with regard to the propensity of the resulting Aβ preparation to form pores in planar lipid bilayers and to kill cells. (DOCX) [file pone.0047261.s010.docx]

## Table S1. Comparison of different Aβ aggregation procedures with regard to the propensity of the resulting Aβ preparation to form pores in planar lipid bilayers and to kill cells.

| **Preparation Method** | **Short description of preparation method** | **Final [Aβ], μM** | **Fraction of experiments that showed ion flux above baseline, % ^a, b^** | | |  | **Cell death, % ^c^** | |
| --- | --- | --- | --- | --- | --- | --- | --- | --- |
|  |  |  | **Control  (no Aβ)** | **Aβ_1-40_** | **Aβ_1-42_** |  | **Aβ_1-40_** | **Aβ_1-42_** |
| A | diH_2_O _0 d_ | 15-25 | 10 ± 10  10 ± 10  10 ± 10  10 ± 10  10 ± 10  10 ± 10 | 20 ± 10 | 15 ± 10 |  | 15 ± 2 (***) | 13 ± 2 (***) |
| A | diH_2_O _1 d_ | 15-25 |  | 35 ± 15 | 30 ± 15 |  | 26 ± 2 (***) | 39 ± 2 (***) |
| A | diH_2_O _2 d_ | 15-25 |  | 55 ± 15 (**) | 40 ± 15 (*) |  | 35 ± 2 (***) | 43 ± 2 (***) |
| A | diH_2_O _3 d_ | 15-25 |  | 50 ± 15 (**) | 25 ± 10 |  | 38 ± 2 (***) | 44 ± 2 (***) |
| A  A | diH_2_O _10 d_  diH_2_O _20 d_ | 15-25  15-25 |  | 30 ± 15  20 ± 10 | 10 ± 10  0 |  | 45 ± 3 (***)  28 ± 3 (***) | 43 ± 3 (***)  36 ± 4 (***) |
| B^d^_GL Bioscience_ | (non-lyophilized Aβ),diH_2_O_0 d_ | 15-25 | 10 ± 10 | 20 ± 15 | 40 ± 15 |  | 22 ± 4 (***) | 40 ± 7 (***) |
| B^d^_Biopeptide_ | (non-lyophilized Aβ),diH_2_O_0 d_ | 15-25 | - | - | - |  | 5 ± 2 (*) | 46 ± 6 (***) |
| C | HFIP/ diH_2_O _2 d_ | 10-25 | 10 ± 10 | 30 ± 15 | 15 ± 10 |  | 31 ± 4 (***) | 40 ± 4 (***) |
| D | proteoliposomes (DOPS) | 1-10 | 30 ± 15 | 65 ± 15 (**) | 80 ± 10 (**) |  | not possible | not possible |
| E | proteoliposomes (positively charged) | 1-10 | 35 ± 10 | 75 ± 10 (**) | 70 ± 15 (*) |  | not possible | not possible |

*^a^* The planar lipid bilayer was composed of DOPS:POPE (1:1) lipids dissolved in *n*-heptane at a lipid concentration of 20 mg mL^-1^.
 *^b^* Errors are standard errors of proportion of success rate in pore formation calculated by S.E. = $\sqrt{p(100-p)/n}$, where *p* is the fraction of observed pore formation (in %) and *n* is the number of planar lipid bilayer experiments. Statistical significance of pore formation compared to the respective control sample is determined by Barnard’s exact test (* *p* < 0.1, and ** *p* < 0.05)
^c^ Cell death was defined as 100%- viability in %; errors are standard errors of the mean. The statistical significance of percentage of cell death compared to the respective control sample was determined by Students’ *t* test (* *p* < 0.1, and *** *p* < 0.01)
^d^ Biopeptide and GL Bioscience indicate two different suppliers of Aβ
