# Supplementary material for: Multivariate Analyses of Amyloid-Beta Oligomer Populations Indicate a Connection between Pore Formation and Cytotoxicity
Source: PLoS One. 2012 Oct 15;7(10):e47261. doi: 10.1371/journal.pone.0047261 (PMC3471831; doi:10.1371/journal.pone.0047261)
Supplement: Table S2 — Deconvolution of CD spectra of Aβin different preparation methods. (DOCX) [file pone.0047261.s011.docx]

## Table S2. Deconvolution of CD spectra of Aβ in different preparation methods.

| **Preparation** | **% Content of secondary structure** | | | |
| --- | --- | --- | --- | --- |
|  | **Helix** | **β-sheet** | **β-turn** | **Unordered** |
| Aβ_1-40_-diH_2_O _0 d_ | 3 | 25 | 14 | 59 |
| Aβ_1-40_-diH_2_O _1 d_ | 3 | 32 | 17 | 48 |
| Aβ_1-40_-diH_2_O _2 d_ | 4 | 34 | 19 | 43 |
| Aβ_1-40_-diH_2_O _3 d_ | 6 | 35 | 19 | 41 |
| Aβ_1-40_-diH_2_O _10 d_ | 4 | 34 | 20 | 42 |
| Aβ_1-40_-diH_2_O _20 d_ | 9 | 43 | 23 | 24 |
| Aβ_1-40_-HFIP/diH_2_O _2 d_ | 5 | 39 | 21 | 35 |
| Aβ_1-42_-diH_2_O _0 d_ | 4 | 27 | 37 | 33 |
| Aβ_1-42_-diH_2_O _1 d_ | 4 | 30 | 41 | 36 |
| Aβ_1-42_-diH_2_O _2 d_ | 5 | 32 | 42 | 37 |
| Aβ_1-42_-diH_2_O _3 d_ | 5 | 45 | 21 | 29 |
| Aβ_1-42_-diH_2_O _10 d_ | 5 | 44 | 22 | 30 |
| Aβ_1-42_-diH_2_O _20 d_ | 9 | 43 | 23 | 29 |
| Aβ_1-42_-HFIP/diH_2_O _2 d_ | 5 | 41 | 21 | 33 |
